# Rehabilitation Utilization for Parkinson’s Disease in Southern Ghana: A Descriptive Cross‐Sectional Survey

**DOI:** 10.1155/padi/1443255

**Published:** 2026-06-30

**Authors:** Mary Wetani Agoriwo, Marianne Unger, Conran Joseph, Erika Franzén

**Affiliations:** ^1^ Department of Health and Rehabilitation Sciences, Stellenbosch University, Cape Town, South Africa, sun.ac.za; ^2^ Department of Physiotherapy and Rehabilitation Sciences, University of Health and Allied Sciences, Ho, Ghana, uhas.edu.gh; ^3^ Department of Neurobiology, Care Sciences and Society, Karolinska Institute, Stockholm, Sweden, ki.se; ^4^ Medical Unit Allied Health Professionals-Theme Women’s Health and Allied Health Professionals, Karolinska University Hospital, Stockholm, Sweden, karolinska.se

**Keywords:** Ghana, Parkinson’s disease, rehabilitation, utilization

## Abstract

**Background:**

There is limited literature describing rehabilitation utilization among the Parkinson’s disease (PD) population across the world and especially in Africa, despite ample evidence and clinical guidelines in support of rehabilitation for persons with PD (PwPD).

**Objectives:**

To describe the characteristics of PwPD, the types of rehabilitation services and treatment parameters used, and the factors associated with rehabilitation utilization in southern Ghana.

**Methods:**

A descriptive cross‐sectional survey was conducted among PwPD receiving care at one primary and two tertiary hospitals selected from southern Ghana. The MDS‐UPDRS Part III, PDQ‐8, and modified ICF Checklist Clinician Form were used to assess motor function, health‐related quality of life, and rehabilitation use. Descriptive and inferential statistics were conducted with significance set at *p* < 0.05.

**Results:**

Seventy‐five PwPD were included, with 61.3% being males. Engagement in physiotherapy and/or gymnasium activities was reported by 40.0% of participants. The most common indication for physiotherapy was gait difficulties. No participant had used occupational therapy or speech therapy. Nonreferral by neurologists and participants’ poor knowledge of rehabilitation benefits and needs were the main reasons for nonuse of rehabilitation. Gait retraining and strengthening exercises were the most common physiotherapy interventions received by 73.7% of the participants. Longer PD duration was associated with physiotherapy utilization. A total of 42.4% of participants discontinued physiotherapy services, primarily due to transportation challenges and high treatment cost.

**Conclusion:**

Rehabilitation services are underutilized by PwPD in southern Ghana due to limited referrals, poor awareness of benefits, and related barriers. This highlights the need for more accessible and integrated services.

## 1. Introduction

Parkinson’s disease (PD) is a progressive condition which has been reported as the second most common neurodegenerative disease after Alzheimer’s disease [1]. It presents with a complex spectrum of motor (resting tremors, rigidity, bradykinesia, poor balance, etc.) and nonmotor (autonomic system, cognitive impairments, and behavioral disorders, etc.) signs and symptoms which affect daily functioning and quality of life [2]. PD is idiopathic, highly prevalent among the elderly and male populations [3], and has no known cure.

In managing PD, levodopa‐based medications and surgical interventions such as deep brain stimulation can help alleviate PD symptoms [4]. However, as PD progresses, persons with PD (PwPD) develop resistance to pharmacological treatment, which can result in freezing of gait, postural instability with falls, dysphagia, cognitive impairments, and increased caregiver burden [5]. Nevertheless, rehabilitation is an essential component of PD management, aimed at reducing disability, restoring function, and improving quality of life [6, 7]. Numerous rehabilitation therapies such as physiotherapy (PT), speech and language therapy (SLT), and occupational therapy (OT) are beneficial in enhancing motor and cognitive functions, activities of daily living, and overall quality of life in PwPD, not only in the short‐term but also over the long‐term [8]. However, PT is mostly used among PwPD, likely due to its availability across all levels of healthcare and health benefits although SLT and OT are also beneficial [9, 10]. For instance, a minimum of 4 weeks of structured gait retraining could have a persistent beneficial effect of up to a year after treatment completion [11]. Additionally, a study that included 237 PwPD found that maintaining regular high activity levels and exercises was associated with slower decline in PD symptoms [12]. This reflects the description of exercise as medicine which needs to be prescribed as an adjunctive therapy during the early stages of the disease trajectory [13]. Langeskov‐Christensen et al. [13] further described exercise as a preventive therapy against PD, a potential symptom‐modifying intervention and an effective modality for symptomatic management. For the effective use of rehabilitation, clinical guidelines have been developed to provide guidance on the use of multiple rehabilitation therapies relevant to PD management. These include the WHO Package of Intervention for Rehabilitation for PD [14], the 2017 NICE guideline [15], the international consensus statement on delivering multidisciplinary rehabilitation care in PD [16], and professionals’ specific guidelines for PT [17, 18], OT [19], and SLT [20]. All these clinical guidelines recommend that PwPD be referred to rehabilitation services, especially during the early stages of the disease, to facilitate comprehensive assessment and provide education to both patients and their families regarding the benefits of these therapies.

Although there is evidence in support of the effectiveness of rehabilitation for PwPD, there is limited literature that describes its use across the world and especially in Africa [9]. A recent scoping review which included 12 studies from the developed countries reported that the use of rehabilitation among PwPD ranged from 0.9% for OT to 62.5% for PT [9]. Also, a survey from a high‐income country, the Czech Republic, recorded that 28% of the PwPD used PT [21] and a hospital‐based study that used medical claims in the Netherlands also reported 58% of the PwPD had used PT over a 3‐year period [22]. In accessing rehabilitation, several factors have been identified as predictors to its use and these include specific functioning problems with gait, transfers, swallowing and communication, history of falls, female gender, older age, advanced disease stage, high levodopa equivalent dose (≥ 800), and care by a neurologist [9]. The financial burden of living with a degenerative chronic disease is also reported to predict rehabilitation‐seeking behaviors [23, 24].

In Africa, rehabilitation services in general are either unavailable or unaffordable [25, 26]. To support the WHO’s Rehabilitation 2030 and the Universal Health Coverage initiatives, which advocate for integrating rehabilitation into all levels of healthcare and improving services for PwPD, a clearer understanding of rehabilitation service utilization is essential to inform local policies [27, 28]. Given the absence of published data on rehabilitation service utilization among PwPD in Africa [9], it was deemed appropriate to conduct a survey in Ghana to better understand which services are utilized and how they are delivered. Within the Ghana public health system, rehabilitation services such as PT, OT, and SLT are available upon referral by a medical practitioner [10]. Therefore, the specific objectives of this study were to (a) describe the characteristics of PwPD in the southern sector of Ghana, (b) describe the types of rehabilitation services and treatment parameters (such as frequency and number of sessions and treatment duration) utilized, and (c) determine the factors associated with the use of rehabilitation.

## 2. Methods

### 2.1. Study Design and Setting Description

A descriptive cross‐sectional design was used to conduct this study at two teaching hospitals (THs) and one primary healthcare facility (PF) purposefully selected from the southern sector of Ghana as these are deemed representative of services across Ghana. These selected healthcare facilities run specialized outpatient neurology and PD‐specific clinics. One of the teaching hospitals (TH1) is the largest teaching and referral hospital in Ghana with 2000‐bed capacity, located in the national capital of Ghana [29, 30] while the other is a 240‐bed capacity hospital (TH2) located in a regional capital [31]. The neurology clinics run in these THs manage PD and other adult neurological conditions. The PF is a district‐level facility with 70‐bed capacity located in a rural setting. It is the only PF that provides PD‐specific services in Ghana. The Consensus‐Based Checklist for Reporting of Survey Studies (CROSS) was used to guide reporting [32] (Supporting Information 1).

### 2.2. Study Population and Sampling

All PwPD receiving care at the selected healthcare facilities were potentially eligible to participate in the study. To be included, however, they had to have a confirmed diagnosis of idiopathic PD based on either the UK PD Society Brain Bank criteria for diagnoses [33] or the International Parkinson and Movement Disorder Society (MDS) clinical diagnostic criteria [34]. PD diagnosis was confirmed by a neurologist. All adults with PD at any disease stage and with a mini‐mental state examination (MMSE) score of more than 24 were considered for inclusion (permission granted by PAR to use the MMSE scale). The MMSE has not been validated for the Ghanaian population but it demonstrated to be sensitive in identifying cognitive impairment among an elderly population in a clinic‐based study in Ghana [35]. Patients with atypical and secondary Parkinsonism were excluded. On average, 15, 5, and 10 PwPD attended the TH1, TH2, and PF clinics, respectively, per month. Data collection was planned to take place within three months at each study site resulting in a total population of 90 PwPD. The study sample was then estimated at *n* = 74 using Yamane’s formula (*n* = *N*/(1 + Ne^2^), where *N* = population (90) and *e* = 0.05, at 95% confidence interval):

(1) n=N1+Ne2=90190+ 0.052=9010.225+=73.4.

Based on the monthly clinic attendance, quotas were allocated to each study site, 50% of the study sample from TH1, 12.3% from TH2, and 24.7% from PF. A convenient sampling method was used to recruit the eligible PwPD into the study. Overall, data collection occurred from March to July 2024.

### 2.3. Instrument for Data Collection

The “Brief Health Information” section of the ICF Checklist Clinician Form [36] was modified to include questions on the use of rehabilitation services on outpatient basis. Demographic details of participants (age, gender, employment, duration of PD, etc.) and their PD medications and dosages were collated (Supporting Information 1). In addition, two standard clinical tools, the International Parkinson and Movement Disorder Society‐Unified Parkinson’s Disease Rating Scale (MDS‐UPDRS) Part III and the Parkinson’s Disease Questionnaire (PDQ‐8), were used for data collection.

The MDS‐UPDRS Part III [37] was used to assess the level of motor function of the PwPD. Permission was obtained from the MDS before using the tool. The principal investigator (first author) has over a decade experience in managing PD and underwent training and was licensed to conduct motor examination on PwPD with the MDS‐UPDRS Part III. The PDQ‐8 was used to assess participants’ level of quality of life [38]. The PDQ‐8 is a short version of the PDQ‐39 [38]. It is an 8‐item tool which measures the global impact of PD on the health status of the affected person on eight domains. These include mobility, activities of daily living, emotional well‐being, stigma, social support, cognition, communication, and bodily discomfort. The PDQ‐8 has been reported to be valid and reliable in comparison to the PDQ‐39 with an internal reliability of *α* = 0.80 and a strong correlation of *r* = 0.93, *p* < 0.001 [39], and *r* = 0.98, *p* < 0.001 [38]. The questions have a five‐point Likert scale response given as 0–never, 1–occasionally, 2–sometimes, 3–often, and 4–always. A license was obtained from the Clinical Outcomes at Oxford University Innovation prior to use.

### 2.4. Piloting of Questionnaires

The questionnaires were piloted to ensure the feasibility of the processes and administration underlying the study as well as understanding of the questions by the Ghanaian population. The pilot was conducted at the neurology clinics of a TH and a PT department that were not included as study sites. Eight PwPD receiving care at these facilities were recruited for the pilot. Efforts were made to include persons with varying age, gender, disease severity and duration, and level of education. For demographic details of pilot participants, see Supporting Information 3.

### 2.5. Participant Recruitment

Posters were placed at the neurology/PD clinics of the selected facilities to create awareness about the study. PwPD were engaged by the nurses who run the clinics and those who expressed interest in participating in the research were referred to the researcher for recruitment. Participants were given the study information sheet and their contact phone numbers were recorded for follow‐up calls to discuss and answer any further questions concerning the study.

### 2.6. Procedure for Data Collection

A favorable time and day for data collection were agreed upon by the participant and researcher. The venue for data collection was scheduled for either participants’ homes or at the neurology/PD clinic. However, the researcher ensured that the venue was spacious enough (about 10 m or more space available) to allow room for the gait test component of the survey. Safety measures were also put in place to prevent falls during the physical assessment. Data collection was carried out in both “ON” and “OFF” states. Thus, participants’ perceived best or worst moments of functioning after medication intake were recorded [37]. Prior to the data collection, the study’s aims and objectives were again explained, and a written informed consent was obtained. The survey and physical assessment were conducted by the researcher. For participants who could not understand English, the questions were interpreted in the local dialect (Twi or Ewe). It took approximately 20–30 min to complete the survey for each participant. Data collection was conducted by MWA, who is a physiotherapist and has lived and worked in the study country.

### 2.7. Data Processing and Analysis

Anonymized data were imported onto an Excel spreadsheet and analyzed using the statistical package for social sciences (SPSS) version 29.0. Descriptive statistical analysis was performed for all data on demographic, rehabilitation use, MDS‐UPDRS Part III, and PDQ‐8. Findings were presented as frequencies, percentages, and means with standard deviation and illustrated with tables and charts. The sum of each participants’ MDS‐UPDRS Part III scores was calculated and rated as mild (≤ 32); moderate (33–58); and severe (≥ 59) limitations in motor performance based on the standard categorization and cut offs of the tool [40].

The levodopa equivalent daily dose (LEDD) was calculated for each participant’s PD medication using an online LEDD calculator [41] and interpreted based on guidance provided in literature [42, 43]. The two participants who were on Mucuna were excluded from the LEDD calculation since this is not captured on the online calculator. They were only on Mucuna.

A cumulative summary index (SI) of the eight items PDQ‐8 was calculated on a scale ranging from 0 (perfect health) to a maximum score of 100% (worse health). However, based on the categorization of the MDS‐UPDRS scores, the PDQ‐8 summary index (PDQ‐8SI) scores were further categorized into three groups as perfect health (≤ 24%), moderate health (25%–44%), and worse health (≥ 45%). The PDQ‐8SI was derived by summing up the scores of the eight items after they are converted into percentages. The summed‐up scores were then divided by eight to give a single SI value which is the PDQSI. Data normality was tested with the Shapiro–Wilks test. The MDS‐UPDRS Part III data set was normally distributed across the study sites while the PDQ‐8 data set was not normally distributed. Therefore, the differences among participants across the three study sites regarding the MDS‐UPDRS Part III were assessed with one‐way ANOVA and the PDQ‐8 was assessed with the Kruskal–Wallis (H) test. The difference in MDS‐UPDRS Part III scores between participants who used or did not use rehabilitation and participants in the “On” or “Off” state were assessed with the independent *t*‐test. The difference in PDQ‐8 scores between participants who used or did not use rehabilitation was assessed with Mann–Whitney *U* test. Logistic regression analysis was conducted for the binary outcome on use (yes/no) of rehabilitation. Univariate logistic regression analysis was performed to estimate the odds ratios to assess factors (gender, age, marital status, education level, PD duration and stage, PDQ‐8, MDS‐UPDRS Part III, LEDD, and assistive device use) that were associated with the use of rehabilitation. These ten variables were selected based on predictors and nonpredictors for rehabilitation utilization reported in the existing literature [9]. Only variables with *p*‐values less than 0.2 were moved into the multivariate logistic regression analysis to estimate the adjusted odds ratios (AOR). Significance was set at *p* < 0.05.

### 2.8. Ethical Considerations

The study proposal was reviewed and ethical approval was received from the Stellenbosch University Health Research Ethics Committee (S22/11/256 (PhD)), the institutional ethics review boards of the THs (KBTH‐IRB 00087/2023; CCTHERC/EC/2023/152; HTH‐REC (06) FC_2023), and the Ghana Health Service Ethics Review Board (GHS‐ERC:014/05/23). Permission was obtained from the management of the healthcare facilities included. Prior to data collection, informed consent was obtained from the participants and anonymity was assured. No data were collected on the participants’ clinic day to avoid any confusion between usual care received at the clinic and the study. The study procedure was explained, and it was made clear to participants that there was no therapeutic benefit. However, deemed appropriate referrals were made.

## 3. Results

### 3.1. Participant Recruitment and Response Rate

**FIGURE 1:**
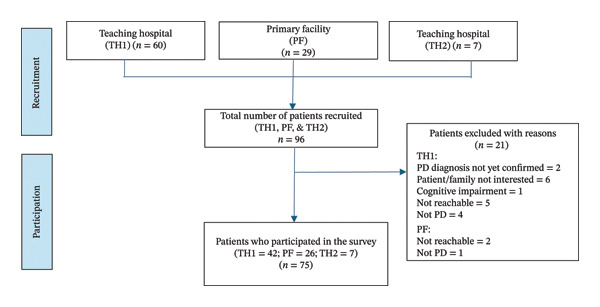
Flowchart for patient recruitment. PD: Parkinson’s disease; Not reachable: These are patients who did not return to the clinic again after recruitment or had their phone numbers persistently not available or out of coverage area or never picked or returned a call.

A 78% (*n* = 75/96) response rate of PwPD was reported with most participants attending the TH in the national capital (Figure 1).

### 3.2. Demographic and Clinical Details of Participants

At the time of data collection, 82.7% of the participants were in the “ON” state while 17.3% of the participants were in the “OFF” state. The majority of the participants (9.3%) in the “OFF” state were from the PF. Table 1 shows the demographic and clinical details of the PwPD across the study sites. Most of the PwPD were males (*n* = 46/75, 61.3%) and the overall mean (SD) age was 66.8 (9.6) years. The mean (SD) disease duration was 5.96 (4.9) and 56.0% were diagnosed within the last 5 years. Most of the participants were in H&Y stage II (*n* = 47/75, 62.7%).

**TABLE 1:** Demographic and clinical details of participants.

Items		Overall *n* = 75 (100)	TH1 *n* = 42 (56.0)	PF *n* = 26 (34.7)	TH2 *n* = 7 (9.3)
Gender	Male	46 (61.3)	23 (30.7)	17 (22.7)	6 (8.0)
	Female	29 (38.7)	19 (25.3)	9 (12.0)	1 (1.3)

Age, years	Mean (SD)	66.8 (9.6)	66.7 (8.5)	67.4 (11.9)	66.0 (7.3)
	Min–max	43–90	52–90	43–89	55–77

Level of education	No formal education	10 (13.3)	2 (2.7)	8 (10.7)	0
	Junior high school	19 (25.3)	12 (16.0)	7 (9.3)	0
	Senior high school	18 (24.0)	11 (14.7)	5 (6.7)	2 (2.7)
	‘O’‐level	6 (8.0)	5 (6.7)	1 (1.3)	0
	Tertiary education	22 (29.3)	12 (16.0)	5 (6.7)	5 (6.7)

Marital status	Never married	3 (4.0)	1 (1.3)	2 (2.7)	0
	Currently married	40 (53.3)	27 (36.0)	9 (12.0)	4 (5.3)
	Separated	4 (5.3)	2 (2.7)	1 (3.8)	1 (1.3)
	Divorced	10 (13.3)	4 (5.3)	4 (5.3)	2 (2.7)
	Widowed	18 (24.0)	8 (10.7)	10 (13.3)	0

Current occupation	Paid employment	3 (4.0)	2 (2.7)	0	1 (1.3)
	Self‐employed	8 (10.7)	6 (8.0)	2 (7.7)	0
	Nonpaid work, such as volunteer/charity	2 (2.7)	0	1 (1.3)	1 (1.3)
	Unemployed (health reason)	17 (22.7)	7 (9.3)	9 (12.0)	1 (1.3)
	Unemployed (other reasons)	1 (1.3)	1 (1.3)	0	0
	Retired	44 (58.7)	26 (34.7)	14 (18.7)	4 (5.3)

MMSE score	Mean (SD)	27.8 (1.8)	28.1 (1.7)	27.1 (1.9)	28.9 (0.7)
	Min–max	24–30	24–30	24–30	28–30

Participant state	‘On’ state	62 (82.7)	37 (49.3)	19 (25.3)	6 (8.0)
	‘Off’ state	13 (17.3)	5 (6.7)	7 (9.3)	1 (1.3)

H&Y stages	II	47 (62.7)	28 (37.3)	14 (18.7)	5 (6.7)
	III	16 (21.3)	9 (12.0)	6 (8.0)	1 (1.3)
	IV	10 (13.3)	3 (4.0)	6 (8.0)	1 (1.3)
	V	2 (2.7)	2 (4.8)	0	0

PD duration, years	Mean (SD)	5.96 (4.94)	5.47 (4.50)	6.51 (5.67)	6.87 (5.03)
	Min–max	0.08–20.00	0.08–19.00	0.08–20.00	1.10–16.00
	< 5	42 (56.0)	24 (32.0)	15 (20.0)	3 (4.0)
	6–10	19 (25.3)	11 (14.7)	5 (6.7)	3 (4.0)
	> 10	14 (18.7)	7 (9.3)	6 (8.0)	1 (1.3)

Current PD medication	Levodopa/benserazide	45 (60.0)	20 (26.7)	24 (32.0)	1 (1.3)
	Levodopa/carbidopa	26 (34.7)	21 (28.0)	0	5 (6.7)
	Stalevo	1 (1.3)	1 (1.3)	0	0
	Mucuna	2 (2.7)	0	2 (2.7)	0
	No medication	1 (1.3)	0	0	1 (1.3)

Additional medication	Trihexyphenidyl	20 (26.7)	18 (24.0)	1 (1.3)	1 (1.3)
	Amantadine	6 (8.0)	5 (6.7)	0	1 (1.3)

LEDD (mg)	Mean (SD)	579.1 (270.3)	570.1 (287.9)	627.1 (238.6)	450 (252.9)
	Min–max	200–1570	200–1570	200–1000	300–950

MDS‐UPDRS Part III	Mean (SD)	40.0 (18.1)	34.90 (16.3)	49.08 (17.3)	36.9 (18.7)
	Min–max	7–79	7–76	13–79	16–68
	Mild limitation (≤ 32)	30 (40.0)	23 (30.7)	3 (4.0)	4 (5.3)
	Moderate limitation (33–58)	31 (41.3)	15 (20.0)	15 (20.0)	1 (1.3)
	Severe limitation (≥ 59)	14 (18.7)	4 (5.3)	8 (10.7)	2 (2.7)

PDQ‐8SI	Mean (SD)	35.0 (18.8)	32.1 (18.2)	40.8 (17.6)	30.8 (24.0)
	Min–max (%)	3.1–68.8	6.3–65.6	3.1–68.8	6.3–68.8
	Perfect health (≤ 24%)	23 (30.7)	16 (21.3)	4 (5.3)	3 (42.9)
	Moderate health (25%–44%)	27 (36.0)	13 (17.3)	12 (16.0)	2 (2.7)
	Worse health (≥ 45%)	25 (33.3)	13 (17.3)	10 (13.3)	2 (2.7)

Comorbidities	No medical condition	24 (32.0)	9 (12.0)	14 (18.7)	1 (1.3)
	Hypertension	37 (49.3)	24 (32.0)	9 (12.0)	4 (5.3)
	Diabetes	9 (12.0)	7 (9.3)	2 (2.7)	0
	Glaucoma	4 (5.3)	4 (5.3)	0	0
	High blood cholesterol	4 (5.3)	4 (5.3)	0	0
	BPH	9 (12.0)	7 (9.3)	1 (1.3)	1 (1.3)
	Spine problems	4 (5.3)	2 (2.7)	0	2 (2.7)
	Osteoarthritis	2 (2.7)	1 (1.3)	1 (1.3)	0
	Foot ulcer	2 (2.7)	1 (1.3)	0	1 (1.3)
	Others	8 (10.7)	6 (8.0)	0	2 (2.7)

Use of assistive devices	No	43 (57.3)	28 (37.3)	13 (17.3)	2 (2.7)
	Spectacles	11 (14.7)	8 (10.7)	2 (2.7)	1 (14.3)
	Walking stick	17 (22.7)	6 (8.0)	8 (10.7)	3 (4.0)
	Tetrapod	2 (2.7)	2 (2.7)	0	0
	Elbow crutch	1 (1.3)	1 (2.4)	0	0
	Zimmer frame	3 (4.0)	1 (1.3)	1 (1.3)	1 (1.3)
	Wheelchair	2 (2.7)	0	1 (1.3)	1 (1.3)

*Note:* Spine problems: degenerative spine disease, scoliosis, lumbar stenosis, and spondylosis; others (one each): G6PD deficiency, hypotension, cataract, sexual dysfunction, asthma, hyperthyroidism, burning sensation in the abdomen and foot, and stroke; numbers in brackets are reported in percentages, except for the mean (SD).

Abbreviations: BPH = benign prostatic hyperplasia; H&Y = Hoehn and Yahr (no stage I recorded); LEDD = levodopa equivalent daily dose; min–max = minimum–maximum; MMSE = mini‐mental state examination; PD = Parkinson’s disease; PF = primary facility; SD = standard deviation; TH = teaching hospital.

Types of PD medications used were significantly different (*p* < 0.001) among the PwPD across the study sites and the most common medication used was levodopa/benserazide (*n* = 40/75, 60.0%). Trihexyphenidyl was the most common additional medication used by the participants. The overall mean (SD) LEDD was 579.1 (270.3) mg. The overall mean (SD) score of the MDS‐UPDRS Part III was 40.0 (18.1), and comparing the mean scores across the study sites, participants from PF had significantly worse motor function compared to TH1 and TH2 participants (one‐way ANOVA, *F* = 5.74, *p* = 0.005). Overall, the mean (SD) PDQ‐8SI score was 35.0 (18.8) and there was no significant difference (Kruskal–Wallis, *H* = 3.38, *p* = 0.19) in the mean scores across the study sites. Hypertension was the most frequently reported co‐morbidity (*n* = 37/75, 49.3%). More than half of the participants (*n* = 43/75, 57.3%) did not use any assistive devices for walking. The most common walking aid used by participants was a walking stick (*n* = 17/75, 22.7%) and the assistive devices were mostly self‐prescribed (*n* = 16/75, 21.3%).

### 3.3. Use of Rehabilitation

#### 3.3.1. Rehabilitation Use, Source, and Indication for Referral

**FIGURE 2:**
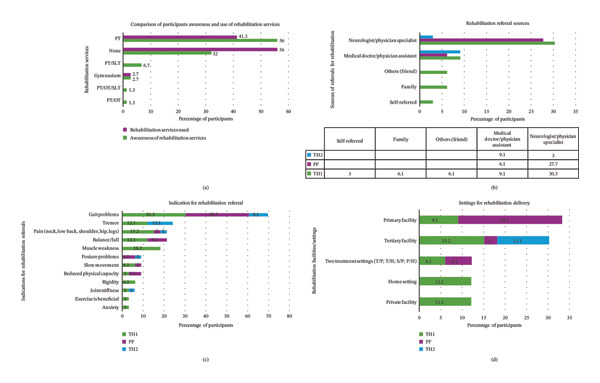
Rehabilitation use, settings, sources, and indications for referral. TH: teaching hospital; PF: primary facility; PT: physiotherapy; SLT: speech and language therapy; OT: occupational therapy; T/P: tertiary/private; T/H: tertiary/home; S/P: secondary/primary; P/H: primary/home.

Overall, 68.0% (*n* = 51/75) of the participants were aware of PT, OT, SLT, or gymnasium activities as means of rehabilitation interventions. Figure 2a illustrates a comparison of awareness and use of rehabilitation among PwPD. Only 44% (*n* = 33/75) of the participants had used rehabilitation services, 41.3% used PT [TH1 (21.3%); PF (14.7%); TH2 (5.3%] and 2.7% from TH1 engaged in gymnasium activities. Persons with Hoehn and Yahr stage II PD were the majority (*n* = 21/33, 63.6%) who used PT. None of the participants assessed OT or SLT services, although 29.3% (22/75) reported mild (19/75) to moderate (3/75) speech problems, as measured by the item 3.1 (speech) of the MDS‐UPDRS Part III. The main reasons reported by the 42 (56.0%) PwPD [TH1 (32.0%); PF (20.0%); TH2 (4.0%)] who did not use any rehabilitation service were that their neurologist had not referred them (*n* = 38/42, 90.5%) [TH1 (50.0%); PF (35.7%); TH2 (4.8%)], they had no knowledge of the benefits (*n* = 21/42, 50.0%) [TH1 (35.7%); PF (11.9%); TH2 (2.4%)], or they saw no need (*n* = 10/42, 23.8%) [TH1] for rehabilitation. Other reasons reported by participants from TH1 were that rehabilitation was costly (4.8%) and they did not have time for rehabilitation (4.8%), and 1 (2.4%) of the participants from TH2 indicated that there were no rehabilitation professionals in their locality.

Many of the participants (*n* = 20/33, 60.6%) who used rehabilitation (i.e., PT) received referrals from the neurologist or physician specialist and the percentage distribution across the study sites is shown in Figure 2b. Referral for PT was mainly determined by the patients’ reported functioning problems. The most common indication for referral to PT reported by participants was gait problems (69.7%) and the study site distribution is shown in Figure 2c. Other common indications were tremor (24.2%), balance/fall problems (21.2%), pain (21.2%), and muscle weakness (18.2%). The participants commonly received rehabilitation services at primary (33.3%) or tertiary (30.3%) healthcare facilities (Figure 2d).

### 3.4. State of Rehabilitation Use

#### 3.4.1. Participants Who Stopped Rehabilitation

At the time of data collection, 42.4% (*n* = 14/33) of the participants across the study sites who had used PT for a period ranging from 1 week to 2 years since their diagnosis had stopped using the services. These participants who discontinued had received an overall median (min–max) of 19 (1–204) PT sessions over an overall median duration of 4.5 (0.25–24) months. Most of these participants (*n* = 10/14, 71.4%) received the rehabilitation service (PT) for 6 months and discontinued. The maximum duration for rehabilitation before discontinuing was 2 years. The key reasons for the discontinuation were transportation challenges (*n* = 7/14, 50.0%) and treatment costs (*n* = 6/14, 42.9%). Other reasons reported were recovery expectations not met, poor family support, worsening of comorbidities, prolonged waiting time, no carer to accompany patient, discharged from PT, physiotherapist no longer available, patient relocated, and patient in pain resulting from other comorbidities.

#### 3.4.2. Participants Still in Rehabilitation

Only participants from TH1 and PF were still undergoing rehabilitation (PT) at the time of data collection (Table 2). The median number of treatment sessions recorded was 4 with a minimum of 1 session and a maximum of 780 sessions. The median duration was 4 months and ranged from 0.25 to 60 months. The majority (*n* = 12/19, 63.2%) of participants who were still having PT had received treatment for 6 months or less since their diagnosis. PwPD mostly receive treatment sessions once a week (26.3%) or once a month (36.8%). Most of the participants had performed 1–3 activities (e.g., treadmill walking and squatting) for rehabilitation (*n* = 14/19, 73.7%) and received individualized treatment (*n* = 15/19, 78.9%).

**TABLE 2:** Rehabilitation (physiotherapy) treatment parameters.

Items		Overall (participants still having PT) *n* = 19 (25.3)	TH1 *n* = 10 (13.3)	PF *n* = 9 (12.0)
Number of sessions	Median (min–max)	4 (1–780)	26.5 (2–780)	2 (1–50)
	≤ 10	11 (57.9)	4 (21.1)	7 (36.8)
	21–50	4 (21.1)	2 (10.5)	2 (10.5)
	> 100	4 (21.1)	4 (21.1)	0

Treatment duration (months)	Median (min–max)	4 (0.25–60)	8 (0.75–60)	3 (0.25–12)
	≤ 1	5 (26.3)	2 (10.5)	3 (15.8)
	2–6	7 (36.8)	3 (15.8)	4 (21.1)
	12	3 (15.8)	1 (5.3)	2 (10.5)
	24	3 (15.8)	3 (15.8)	
	60	1 (5.3)	1 (5.3)	

Frequency of sessions	Once a week	5 (26.3)	3 (15.8)	2 (10.5)
	2 ×/week	2 (10.5)	2 (10.5)	0
	3 ×/week	3 (15.8)	3 (15.8)	0
	5 ×/week	1 (5.3)	1 (5.3)	0
	Once a month	7 (36.8)	0	7 (36.8)
	Every 6 months	1 (5.3)	1 (5.3)	0

Treatment duration (minutes)	30–45	10 (52.6)	3 (15.8)	7 (36.8)
	60	9 (47.4)	7 (36.8)	2 (10.5)

Number of treatment activities	1–2	7 (36.8)	3 (15.8)	4 (21.1)
	3	7 (36.8)	3 (15.8)	4 (21.1)
	4–5	4 (21.1)	3 (15.8)	1 (5.3)
	≥ 6	1 (5.3)	1 (5.3)	0

Form of treatment	Individual	15 (78.9)	10 (52.6)	5 (26.3)
	Group	2 (10.5)	0	2 (10.5)
	Combined	2 (10.5)	0	2 (10.5)

*Note:* Numbers in brackets are reported as percentages, except for the median (min–max).

Abbreviations: Min–max = minimum–maximum; PF = primary facility; TH = teaching hospital.

The most common PT interventions received by the PwPD at the PT departments were gait retraining (overground or treadmill walking) (*n* = 14/19, 73.7%); strengthening exercises (squats, dumbbells, straight leg raises (SLR), sit‐to‐stand, and stair climbing) (*n* = 14/19, 73.7%); flexibility exercises (stretches, ROM exercises, and ergonomic cycling) (*n* = 11/19, 57.9%); balance training and postural correction (*n* = 6/19, 31.6%); and massage (*n* = 5/19, 26.3%). A few of the participants reported on the use of electrotherapy (transcutaneous electrical nerve stimulation (TENS)) (15.8%), hot pack to lower back (10.5%), and deep breathing exercise (5.3%). For home exercises, 36% (*n* = 27/75) of the participants had no routine home activities while 64% (*n* = 48/75) performed some routine activities at home. The most common home activity was gait retraining (*n* = 43/75, 57.3%), mainly overground walking and treadmill walking for one PwPD with personal treadmill at home. Other activities performed included flexibility exercises (stretches) (9.3%); strengthening exercises (dumbbells, sit‐to‐stand, stair climbing, and pulley) (8.0%); singing (4.0%); and other activities such as routine ADLs, dancing, reading, posture correction with a rod, breathing exercises, and balance exercises.

### 3.5. Differences in Participants’ Characteristics on Use and Nonuse of Rehabilitation (PT) and Factors Associated With PT Use

Regarding how long participants had lived with PD (PD duration), those who had longer PD duration significantly used PT compared to those with shorter PD duration (Kruskal–Wallis test, *H* = 10.70; *p* = 0.001). In addition, there was no statistically significant difference (MDS‐UPDRS Part III: *t*‐test, *t* = −1.07, *p* = 0.29; PDQ‐8SI: Mann–Whitney *U* test = 758.5, *p* = 0.48) between the mean (SD) scores on MDS‐UPDRS Part III (42.52 (17.77)) and PDQ‐8SI (36.55 (20.93)) for PwPD who used rehabilitation and those without rehabilitation [MDS‐UPDRS Part III: (38.02 (18.17)); PDQ‐8SI (33.71 (17.08))]. Table 3 shows the full model outcome of the univariate and multivariate regression analysis. PD duration was found to be significantly (*p* = 0.004) associated with the use of PT.

**TABLE 3:** Full model regression analysis for use and nonuse of rehabilitation (PT).

Variables	OR (95% CI)	*p*‐value	AOR (95% CI)	*p*‐value
Age	1.018 (0.970–1.069)	0.461		

Gender Male (ref) Female	0.526 (0.202–1.374)	0.190^a^	0.450 (0.152–1.327)	0.148

Level of education No formal education (ref) Has formal education	2.000 (0.475–8.422)	0.345		

Marital status No partner (ref) Has partner	1.692 (0.672–4.265)	0.265		

H&Y disease stage Nonadvanced (ref) Advanced	0.586 (0.160–2.148)	0.420		

PD duration	1.176 (1.053–1.313)	0.004^a^	1.192 (1.059–1.342)	0.004^b^
PDQ‐8SI	1.008 (0.984–1.033)	0.513		
MDS‐UPDRS Part III	1.014 (0.988–1.041)	0.283		
LEDD	1.001 (0.999–1.003)	0.165^a^	1.001 (0.999–1.003)	0.234
Assistive device use	0.654 (0.260–1.647)	0.367		

*Note:* ref: reference for the logistic regression analysis; has formal education: junior high school, senior high school, ‘O’‐level, and tertiary education; no partner: never married, separated, divorced, and widowed; has partner: currently married; nonadvanced: stage II; advanced: stages III, IV, and V; PDQ‐8SI: Parkinson’s disease questionnaire‐8 summary index; MDS‐UPDRS: International Parkinson and Movement Disorder Society‐Unified Parkinson’s Disease Rating Scale.

Abbreviations: AOR = adjusted odds ratio; CI = confidence interval; H&Y = Hoehn and Yahr; LEDD = levodopa equivalent daily dose; OR = odds ratio; PD = Parkinson’s disease.

^a^Variables with *p* < 0.2 moved into multivariate logistic regression analysis.

^b^Significant variable.

## 4. Discussion

To the best of the authors’ knowledge, this is the first study to assess the use of rehabilitation among PwPD in Ghana. The findings provide indicators and associated factors for the utilization of PT for the management of PD. The study also reveals the main reasons for the nonuse of rehabilitation and discontinuation of PT services.

The study found that only 44% of the participants had used PT or private gymnasium services. This usage aligns with the 0.9%–62.5% rehabilitation use in developed countries [9], but it remains suboptimal given that the majority of the study participants were expected to have utilized rehabilitation for managing their functioning problems right from the time of diagnosis. Additionally, none of the participants utilized SLT and OT services. Unlike PT services, OT and SLT services are not available at all three levels of healthcare [10], which may explain why no participant used these services. Although PT services are available across the healthcare levels in Ghana [10], they are not always near to the patients’ homes. This is what led two of the study participants to use the gymnasium due to proximity. Generally, the access and utilization of rehabilitation, especially SLT and OT, among PwPD across Ghana and Africa at large remain limited due to structural (e.g., infrastructure, professionals, referral, and payment systems) and process‐related (e.g., expertise, interventions, and treatment protocols) challenges [10, 25, 26]. It is imperative to establish these services across all levels of healthcare to increase accessibility. It is equally essential to provide training for gymnasium instructors on foundational knowledge of PD and its management, given that the gymnasiums have increasingly become alternative avenues for physical exercise.

The majority of the participants who utilized PT were mostly referred to the service by a neurologist or a physician specialist, with most presenting at H&Y stage II of the disease. Anecdotally, in Ghana, PwPD often seek healthcare at H&Y stage II, when symptoms have progressed from one side of the body to the other. However, as PD advances and remains without a definitive cure, coupled with other reasons, patients may disengage from healthcare services. This observation may explain the high number of stage II PD participants in this study. Notably, 40% of the study participants who had used PT discontinued the service after about 6 months. The main reasons reported by the study participants for discontinuing the PT services were transportation challenges and high treatment costs. Other factors included unmet recovery expectations, poor family support, absence of a caregiver, worsening comorbidities with accompanying pain, patient relocation, discharge from PT, prolonged waiting time, and unavailable therapists. Irrespective of study site, the PwPD from the tertiary and the primary facilities lived relatively far from PT units, experiencing transportation challenges or incurring substantial transportation costs. Given PD’s chronic nature, persistent symptoms can lead to patient disappointment and caregiver fatigue. This underscores the need for a behavior‐change approach in patient‐centered care emphasizing goal setting, realistic expectations, social support, and practical problem‐solving strategies. It further highlights the significance of early education on the chronic nature of PD, alongside the essential role of sustained care and ongoing rehabilitation.

Furthermore, the study identified gait disturbances, particularly reduced feet clearance and diminished arm swing as the primary reason for referrals to PT services. Other reasons for PT referral included tremor, imbalance/fall problems, pain, and muscle weakness. These findings are consistent with the indications for referral to rehabilitation services found in Agoriwo et al.’s review which included 12 studies from developed countries [9]. While the study findings offer valuable insights, individualized patient assessments remain essential for delivering patient‐centered care. The participants who utilized PT mostly received the services at a primary or tertiary healthcare facility near their homes. The main PT interventions received by the PwPD at the departments were gait and balance training, strengthening and flexibility exercises, postural correction, and massage, which addressed their reasons for seeking PT. These interventions align with the WHO Package of Intervention for Rehabilitation for PD [14] and the American Association of Physical Therapy’s clinical guideline for PT management of PD [17] except massage, although it may be beneficial for managing both motor and nonmotor symptoms [44]. A few participants also reported the use of TENS and hot packs, which are not recommended evidence‐based interventions. This highlights the need for ongoing professional development on evidence‐based practices, such as compensatory interventions (e.g., cueing) relevant for PD management. Most of the PwPD received PT once a week or once a month although Alberts and Rosenfeldt recommend 30–40 min exercise, 3 times per week [45]. This limited frequency of therapy sessions may be attributed to challenges such as transportation and treatment cost mentioned earlier. However, for continuity of treatment, over 60% of the participants engaged in home‐based activities, mostly overground and treadmill walking. Other home exercises were singing, routine ADLs, dancing, reading, posture correction with rod, and balance, flexibility, strengthening, and breathing exercises. This approach aligns with evidence indicating that maintaining physical activity is essential in PD management, as continuous exercise has been shown to reduce the rate of symptom decline [12, 13].

Among the study participants who did not engage in rehabilitation, the primary reasons cited included the absence of referral from the neurologists or medical practitioners, as well as limited awareness of the potential benefits of rehabilitation. Other barriers to rehabilitation use included high cost of rehabilitation, time constraints affecting attendance at rehabilitation appointments, and absence of rehabilitation professionals in participants’ community. This highlights a broader information gap on rehabilitation use among stakeholders. Similar issues such as lack of referral, awareness, SLT, and OT have been reported in Tanzania [25] and across Sub‐Saharan Africa [26, 46]. To address these issues, it is essential to educate PwPD, their families, and caregivers, in addition to offering specialized training for relevant healthcare professionals to encourage early engagement with rehabilitation. It further underscores the need for policies that prioritize the establishment of rehabilitation units, particularly SLT and OT, at all levels of healthcare to improve accessibility and utilization. The integration of self‐management strategies within structured rehabilitation interventions, supported by families and caregivers, may serve to improve the utilization of rehabilitation services and promote patient empowerment.

While previous studies have identified various predictors of rehabilitation utilization, this study found that only long PD duration was significantly associated with PT use among the ten variables modeled. In contrast, Agoriwo et al.’s [9] review reported PD duration as a nonpredictor. This current study found that participants with a longer disease duration had 19% increased chance of utilizing PT services. A longer disease duration may reflect a greater disease severity, potentially indicating increased functional problems that could benefit from PT interventions. These findings highlight the importance of individualized assessments, particularly considering the lack of consensus regarding predictive factors for rehabilitation utilization. Notably, initiating rehabilitation at the time of PD diagnosis may play a key role in mitigating motor and nonmotor complications [6, 16].

For practice, a holistic patient‐centered approach to PD care in Ghana is essential, prioritizing early rehabilitation, improved service delivery, and strengthening of the healthcare system. Policy should support integrated PD management and ongoing professional education, particularly for first‐contact practitioners. Rehabilitation services, particularly SLT and OT, should be made accessible and affordable to all PwPD. Future research should explore the needs, experiences, and satisfaction with rehabilitation use among PwPD and their caregivers.

Although the study findings could serve as a reference for improving the utilization of rehabilitation for PwPD and for future research, there were some limitations. The cross‐sectional study design and convenience sampling method used limit causal inferences and the generalizability of study findings to the larger population. Additionally, the small sample from TH2, which resulted from the unavailability of a neurologist to run the clinic, further affected group comparisons. This facility has only one neurologist who had added academic responsibilities that frequently interrupted the clinic schedule coupled with low patient turn‐out on clinic days. In future, a prospective, longitudinal cohort study is warranted to understand the dependencies between patient characteristics and rehabilitation utilization. The authors also acknowledge the impact of excluding two participants who were on Mucuna from the LEDD calculation on the test of association with rehabilitation use. Hopefully, future studies will improve upon LEDD calculators to include Mucuna.

In conclusion, rehabilitation services remain underutilized in the management of PD in southern Ghana, with PT being the only modality accessed by the study participants. None of the study participants utilized SLT and OT services, and a subset of the participants discontinued PT treatment. The primary barriers to the utilization of rehabilitation services included nonreferral by neurologists and limited patient awareness of the benefits of rehabilitation. High treatment costs and transport difficulties contributed to the discontinuation of PT services. PT interventions targeted specific functioning problems, and longer disease duration was associated with utilizing PT.

## Author Contributions

Mary Wetani Agoriwo, Erika Franzén, Marianne Unger, and Conran Joseph contributed equally to the conceptualization of the study. Data curation: Mary Wetani Agoriwo and Conran Joseph. Investigation: Mary Wetani Agoriwo. Methodology: Mary Wetani Agoriwo, Erika Franzén, Marianne Unger, and Conran Joseph. Supervision: Erika Franzén, Marianne Unger, and Conran Joseph. Writing–original draft: Mary Wetani Agoriwo. Writing–review and editing: Erika Franzén, Marianne Unger, and Conran Joseph.

## Funding

This work was carried out with the support of the following organizations: the Organization for Women in Science for the Developing World (OWSD) and the Swedish International Development Cooperation Agency (Sida) (fund reservation number: 3240378593) for funding the first author’s PhD; Transforming Parkinson’s Care in Africa (TraPCAf) project (NIHR133391) and Stellenbosch University Physiotherapy Division Bursary‐PG Strategic Fund (PSF‐2022) for the first author’s tuition fee; and the Mawazo Fellowship Programme (2023‐1‐03) and the Harry Crossley Foundation for the research grant (HCG‐2024).

## Disclosure

These funders had no influence on the conduct, analysis, and interpretation of study findings.

## Conflicts of Interest

The authors declare no conflicts of interest.

The authors declare that “an unauthorized version of the MMSE was used by the study team without permission, however, this has now been rectified with PAR. The MMSE is a copyrighted instrument and may not be used or reproduced in whole or in part, in any form or language, or by any means without written permission of PAR”.

## Supporting Information

Additional supporting information can be found online in the Supporting Information section.

## Supporting information


**Supporting Information** Supporting Information 1: Checklist for Reporting of Survey Studies (CROSS). The table presents the step‐by‐step approach to conducting and reporting this study based on a standardized guideline. Supporting Information 2: researcher‐developed questionnaire. The table presents the questionnaire designed by the researchers to collect data on the demographic details of participants (age, gender, employment, duration of PD, etc.) and their PD medications and dosages. This will be available upon request from the corresponding author. Supporting Information 3: demographic data of pilot participants. This provides details of the demographic data of the pilot participants. This includes information on gender, age, disease duration, education level, marital status, occupation, and mini‐mental state examination score.

## Data Availability

Data are available upon request from the corresponding author.
